# Supplementary material for: What does it take to learn the rules of RNA base pairing? A lot less than you may think
Source: bioRxiv. 2025 Aug 2:2025.07.31.668042. Preprint. [Version 1] doi: 10.1101/2025.07.31.668042 (PMC12324431; doi:10.1101/2025.07.31.668042)
Supplement: Supplement 1 [file media-1.gz › supplemental_material/src/rscape_v2.6.0.tar.gz!/rscape_v2.6.0/lib/infernal/Manuscripts/banded-cyk/tbl-singlets.doc]

| component *i* | | 1 | 2 | 3 | 4 | 5 | 6 | 7 | 8 |
| --- | --- | --- | --- | --- | --- | --- | --- | --- | --- |
|  | | 0.0851 | 0.0159 | 0.1020 | 0.4160 | 0.0745 | 0.0554 | 0.1184 | 0.1327 |
| \|α\| | | 15.4467 | 154.464 | 180.286 | 5.4562 | 0.2199 | 16.4089 | 13.4592 | 19.9059 |
|  |  |  |  |  |  |  |  |  |  |
| *a* | mean α |  |  |  |  |  |  |  |  |
| A | **0.3951** | 0.0373 | **0.9961** | **0.9787** | **0.3109** | **0.3383** | 0.0375 | 0.0864 | **0.8247** |
| C | **0.1635** | 0.0490 | 0.0015 | 0.0052 | **0.2067** | **0.1782** | **0.8916** | 0.0303 | 0.0493 |
| G | **0.2041** | 0.0220 | 0.0023 | 0.0072 | **0.1751** | **0.2905** | 0.0182 | **0.8313** | 0.0569 |
| U | **0.2372** | **0.8917** | 0.0000 | 0.0090 | **0.3073** | **0.1930** | 0.0527 | 0.0519 | 0.0691 |

Table 4: **Parameters of the 8 component Dirichlet mixture emission prior for singlets.**

$q_{i}$ = mixture coefficient for component *i*. Normalized α values >0.10 are in bold faced type (0.10 was arbitrarily chosen to highlight higher values).
